# Novel lead anchor technique using an active fixation quadripolar left ventricular lead in cardiac resynchronization therapy

**DOI:** 10.1002/ccr3.5332

**Published:** 2022-02-02

**Authors:** Yukihiro Inamura, Osamu Inaba, Akira Sato, Junichi Nitta, Masahiko Goya, Tetsuo Sasano

**Affiliations:** ^1^ Department of Cardiology Japanese Red Cross Saitama Hospital Saitama Japan; ^2^ Department of Cardiology Sakakibara Heart Institute Tokyo Japan; ^3^ Department of Cardiology Tokyo Medical and Dental University Tokyo Japan

**Keywords:** active fixation quadripolar left ventricular lead, anchor technique, cardiac resynchronization therapy, dilated hypertrophic cardiomyopathy, heart failure, left ventricular lead

## Abstract

In this report, we present a case of successful advancement of a LV lead into tortuous vessels. This was achieved by deep engagement of the coronary sinus with a cannulation catheter by applying the anchor technique using the Medtronic Attain Stability Quad lead.

## Graphical abstract

We present the “lead anchor” technique using the Medtronic Attain Stability Quad lead. Fixing the lead temporarily and pulling the fixed lead made the cannulation catheter further. Adjusting the cannulation catheter position could help the lead to advance further.

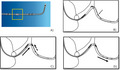

## 1 INTRODUCTION

Cardiac resynchronization therapy (CRT) is an important treatment for patients with heart failure.^1^, ^2^ In clinical practice, it is difficult to advance a left ventricular (LV) lead through narrow or tortuous vessels in some cases. In this report, we present a case of successful advancement of a LV lead into tortuous vessels. This was achieved by deep engagement of the coronary sinus with a cannulation catheter by applying the anchor technique using the Medtronic Attain Stability Quad lead. This useful technique for LV lead delivery has not been previously reported in the literature.

## 2 CASE REPORT

### 2.1 History/examination

A 55‐year‐old man presented with heart failure due to hypertrophic cardiomyopathy in the dilated stage. His electrocardiogram revealed a right bundle branch block with a wide QRS complex (QRS length = 208 ms) and first‐degree atrioventricular block (PR length = 316 ms). He had undergone catheter ablation four times for atrial fibrillation/tachycardia and had taken diuretics and beta‐blockers for the same. Despite the rhythm control and adequate medication therapy, he still had dyspnea (NYHA III) and refractory leg edema. To manage the patient's uncontrolled heart failure, we decided to implant a CRT pacemaker. Written informed consent was obtained from the patient before the CRT pacemaker implantation.

### 2.2 Differential diagnosis, investigation, and treatment

**FIGURE 1:**
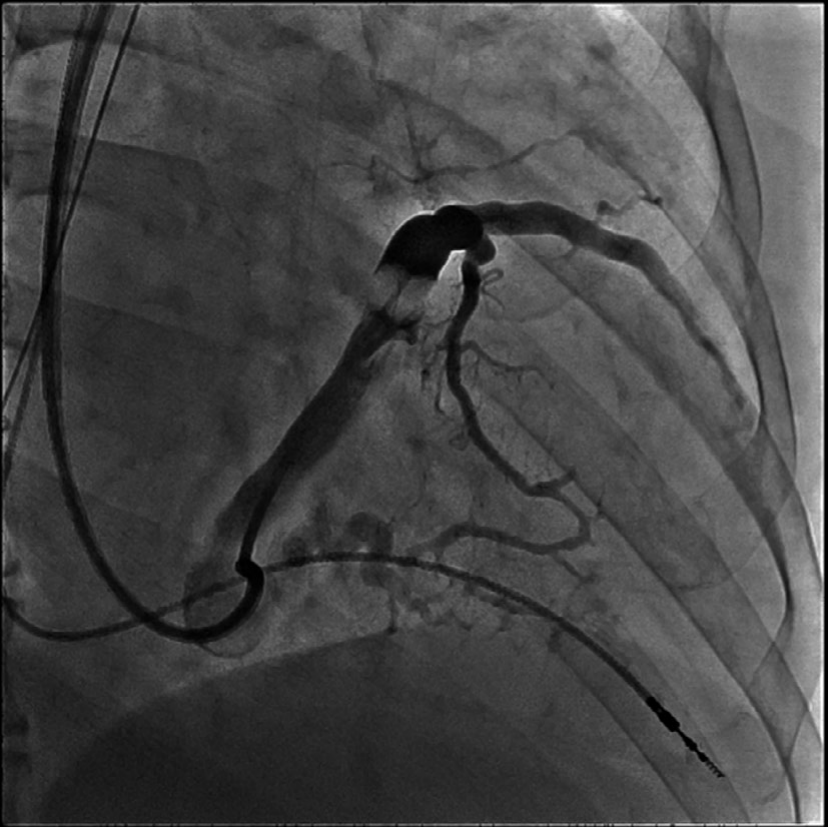
Contrast venography demonstrating a tortuous posterolateral coronary sinus vessel

Angiography of the coronary sinus was used to determine whether the posterolateral vein was suitable to deploy the LV lead. We selected the active fixation LV pacing lead (Attain Stability Quad 4798, Medtronic, Dublin, Ireland) for implantation, which is a quadripolar LV lead with an active fixation helix assembly, designed to position the lead in the coronary sinus.^3^ A large‐curve‐type coronary sinus cannulation catheter (Attain Command + SureValve Integrated Valve, Medtronic, Dublin, Ireland) was used to deploy the lead using a standard over‐the‐wire technique, which involved advancing the pacing lead over the wire into the desired location with the distal electrodes positioned in the mid‐LV segment. However, the lead did not advance smoothly as the target vessel was tortuous (Figure 1). Moreover, the pacing threshold was high, despite the deep insertion of the LV lead. To overcome this, we used an extra support guidewire (Grand Slam, Asahi Intecc, Aichi, Japan) and a 135‐degree subselection catheter (Attain Select II + SureValve subselection catheter, Medtronic); however, we were unable to advance the LV lead further.

Subsequently, we attempted to engage the cannulation catheter deep within the coronary sinus as this could provide better support to pass the LV lead through the tortuous vessel. To achieve this, we applied the anchor technique, which is already reported as a technique for obtaining superior guiding catheter support during the advancement of a balloon catheter in coronary angioplasty.^4^ Once the lead was placed in the deepest position, it was rotated clockwise to achieve active temporary fixation of the lead. Next, we inserted and advanced the cannulation catheter while gently pulling the temporarily fixed LV lead. As the LV lead was fixed, it did not fall out of position despite being pulled, and we were able to advance the cannulation catheter deeper. As a result, superior cannulation catheter support was obtained without the LV lead falling out of position. Following this, the LV lead was rotated counterclockwise, and the fixation mechanism was released from the vein wall. Further, the lead was advanced to the middle lateral position, and refixation was performed.

### 2.3 Outcome and follow‐up

**FIGURE 2:**
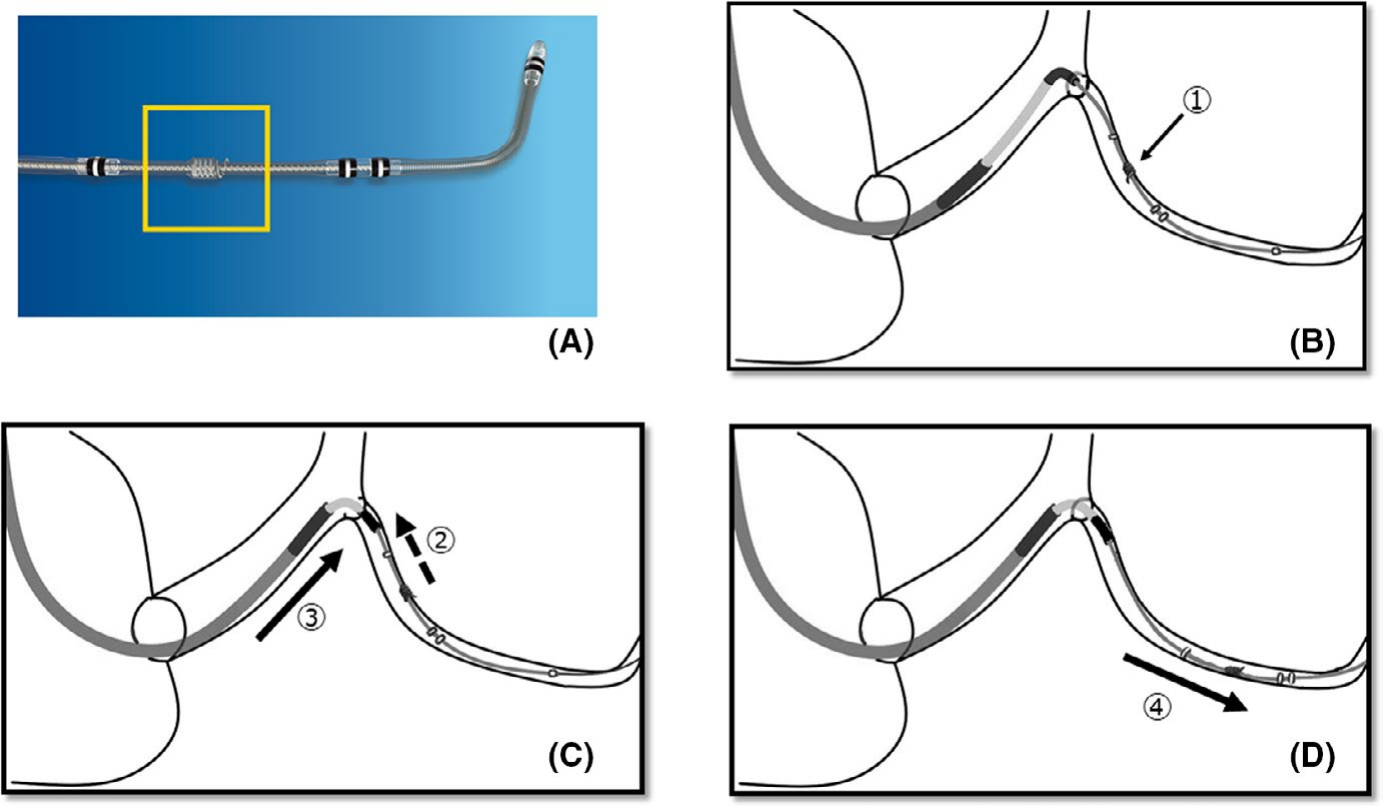
(A) The Medtronic Attain Stability Quad lead. The 5.6‐French non‐electrically active side helix is positioned between the third and fourth electrodes. This picture is provided by Medtronic Japan. (B) Once the LV lead is positioned as deep as possible, it is rotated clockwise and active temporary fixation is performed (arrow ①). (C) Pulling the active fixation lead (arrow ②) while advancing the cannulation catheter deeper (arrow ③). Deep engagement of the cannulation catheter is obtained without the LV lead falling out. (D) After deep engagement, the lead is rotated counterclockwise, fixation mechanism is released from the vein wall, LV lead is advanced to the desired position (arrow ④), and refixation is performed. Deep engagement of the cannulation catheter provides backup support and helps the LV lead to cross the lesion. LV, left ventricular

**FIGURE 3:**
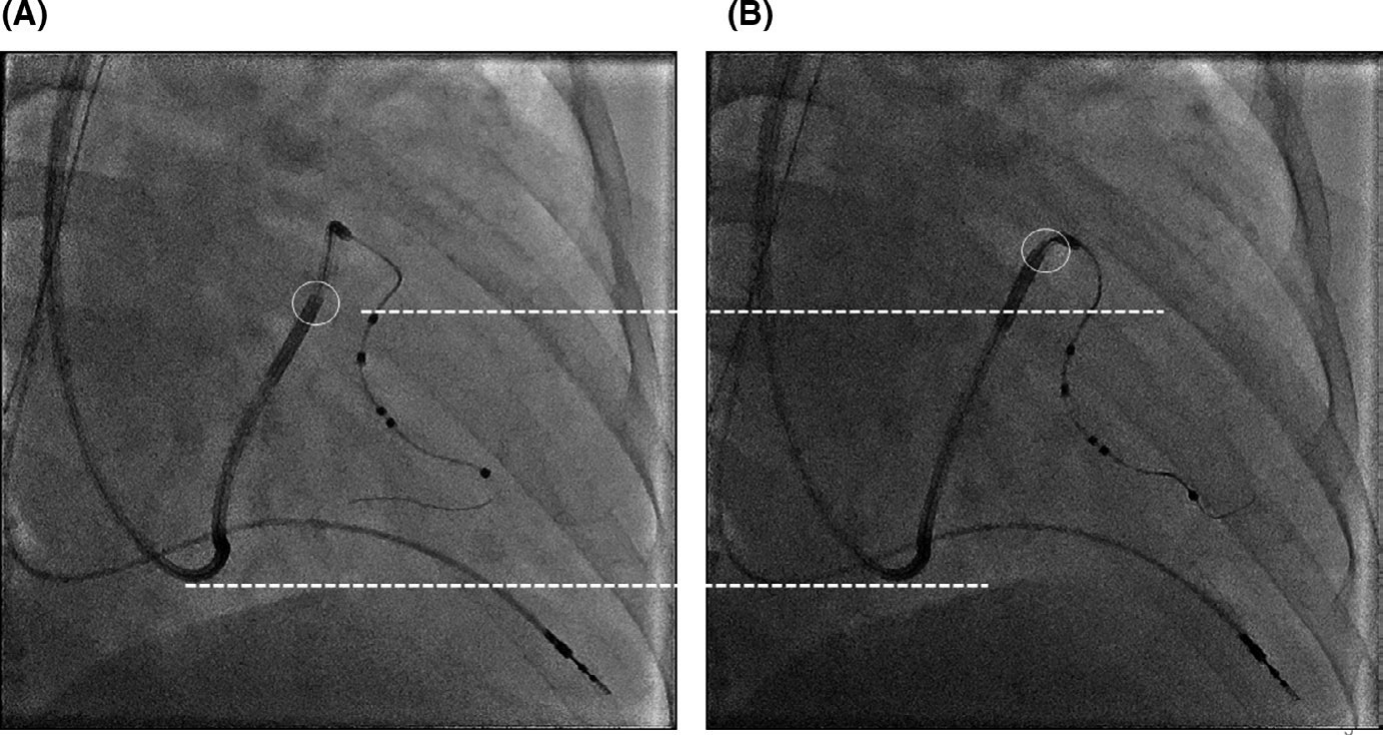
(A) During the “lead anchor technique” is performed. Deep engagement of the cannulation catheter is obtained. (B) After the “lead anchor technique” is performed. The last helix position within the coronary sinus. The pacing threshold was 1.25V/0.4 ms at LV1 to LV2. LV, left ventricular

**FIGURE 4:**
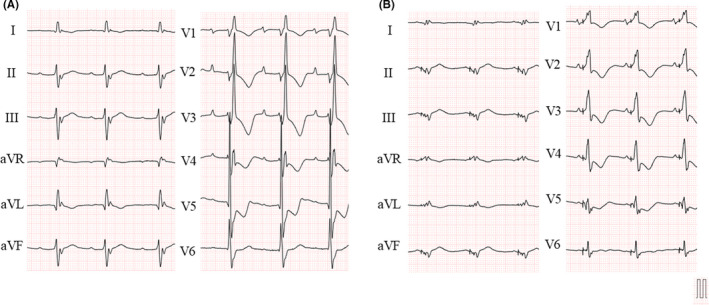
(A) pre‐cardiac resynchronization therapy (CRT) implantation electrocardiogram (ECG). QRS length = 208 ms, PR length = 316 ms. (B) post‐CRT implantation ECG. QRS length = 170 ms

Using this method, the LV lead was successfully and smoothly passed through the tortuous vessel. We called this method the “lead anchor technique” (Figure 2). By obtaining superior cannulation catheter support, we were able to implant the LV lead at a low pacing threshold and without a phrenic nerve stimulation site (Figure 3). After the CRT implantation, the pacing threshold was not increased, and the QRS complex improved to 170 ms (Figure 4). After 9‐month follow‐up, he required hospitalization for heart failure control; however, his dyspnea symptoms reduced and NYHA class had improved (class I−II) upon CRT pacing and treatment with appropriate diuretics.

## 3 DISCUSSION

Cardiac resynchronization therapy relieves symptoms and decreases mortality in patients with heart failure, depressed LV systolic function, and prolonged QRS duration.^5^, ^6^ To obtain maximum benefit from the CRT, it is important to place the LV lead in a suitable region, particularly in lateral and non‐apical positions with a low pacing capture threshold, and without phrenic nerve stimulation. However, lead revision is required due to lead stability issues, such as incorrect pacing location, lead dislodgement, increase in pacing capture threshold, or phrenic nerve stimulation in up to 7% of the CRT implantations.^7^ To overcome these, active fixation LV leads have been developed.^8^ The Medtronic Attain Stability Quad lead, which combines an active fixation mechanism with a quadripolar lead, allows for a targeted approach to LV pacing.^3^, ^9^, ^10^ Using an active fixation lead allows for better stability in the veins, which often have large diameters, compared with a passive fixation lead, the stability of which concerns operators.^11^

It is also important to develop a technique to advance a LV lead through target vessels and to overcome lead stability issues. In clinical practice, it is sometimes difficult to advance a LV lead through narrow and tortuous vessels. In these cases, we use a subselection catheter, extra support guidewire, and a LV lead with minimum French size lead body diameter; however, these devices are sometimes ineffective. In such difficult cases, percutaneous coronary intervention techniques can be useful. For instance, in percutaneous coronary intervention, deep engagement of the guiding catheter could provide good support. Fujita et al. reported the use of the anchor method, in which deep engagement of the guiding catheter was achieved by pulling an anchor balloon inflated in a non‐target vessel. Using this method, superior guiding catheter support was maintained even after balloon deflation,^4^ and Kumagai et al. have reported the balloon anchor method for CRT implantation.^12^ We applied this technique in the LV lead implantation without using a balloon catheter. Temporary fixing of the lead was performed anterior to the target position. Pulling the fixed lead made the cannulation catheter advance further. This method provided superior cannulation catheter support without the LV lead falling out. Adjusting the cannulation catheter position could help the lead to advance further. It should be noted that pulling the lead too hard could cause the fixation helix to stretch. To avoid this, it was important to pull the lead as gently as possible. Moreover, if advancing the cannulation catheter was found to be too difficult, it should not be forced.

To our knowledge, this is the first study that reports the application of the anchor technique for LV lead implantation using the Medtronic Attain Stability Quad lead. The “lead anchor” technique may help in the management of difficult cases of LV lead implantation. This novel technique could help the operators to efficiently advance LV leads through tortuous vessels during the CRT procedure.

## CONFLICT OF INTEREST

All authors have no conflicts of interest to declare.

## AUTHOR CONTRIBUTIONS

Yukihiro Inamura, the corresponding author, drafted this manuscript. Osamu Inaba conceived and designed the manuscript for important intellectual content. Akira Sato provided advice on manuscript design. Junichi Nitta contributed to the discussion of the manuscript and provided advice on manuscript design. Masahiko Goya provided advice on manuscript design. Tetsuo Sasano contributed to the discussion of the manuscript, final approval of the manuscript submitted.

## CONSENT

Written informed consent was obtained from the patient for publication of this case report and accompanying images.

## Data Availability

The data that support the findings of this study are available from the corresponding author upon reasonable request.
